# Ubiquitin‐specific peptidases: Players in bone metabolism

**DOI:** 10.1111/cpr.13444

**Published:** 2023-03-08

**Authors:** Jianlin Shen, Xiaoning Lin, Feifei Dai, Guoli Chen, Haibin Lin, Bangjiang Fang, Huan Liu

**Affiliations:** ^1^ Department of Orthopaedics Affiliated Hospital of Putian University Putian China; ^2^ School of Medicine Putian Universtiy Putian China; ^3^ Department of Emergency Longhua Hospital, Shanghai University of Traditional Chinese Medicine Shanghai China; ^4^ Institute of Emergency and Critical Care Medicine Shanghai University of Traditional Chinese Medicine Shanghai China

## Abstract

Osteoporosis is an ageing‐related disease, that has become a major public health problem and its pathogenesis has not yet been fully elucidated. Substantial evidence suggests a strong link between overall age‐related disease progression and epigenetic modifications throughout the life cycle. As an important epigenetic modification, ubiquitination is extensively involved in various physiological processes, and its role in bone metabolism has attracted increasing attention. Ubiquitination can be reversed by deubiquitinases, which counteract protein ubiquitination degradation. As the largest and most structurally diverse cysteinase family of deubiquitinating enzymes, ubiquitin‐specific proteases (USPs), comprising the largest and most structurally diverse cysteine kinase family of deubiquitinating enzymes, have been found to be important players in maintaining the balance between bone formation and resorption. The aim of this review is to explore recent findings highlighting the regulatory functions of USPs in bone metabolism and provide insight into the molecular mechanisms governing their actions during bone loss. An in‐deep understanding of USPs‐mediated regulation of bone formation and bone resorption will provide a scientific rationale for the discovery and development of novel USP‐targeted therapeutic strategies for osteoporosis.

## Graphical abstract

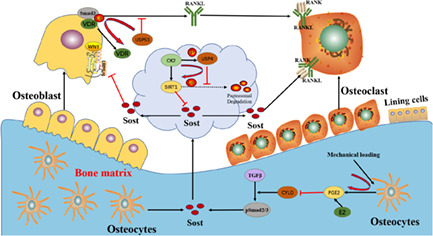

## 1 INTRODUCTION

The ubiquitin–proteasome system (UPS) is an important mechanism that mediates posttranslational modification and protein homeostasis by regulating a variety of cellular activities, including bone metabolism, gene transcription, cell cycle regulation, immune responses, cell receptor function, tumour growth, and inflammatory processes, through the polyubiquitination and degradation of substrate proteins.^1^, ^2^ Proteins are ubiquitinated at least once throughout their life span by the ubiquitin ligase system, which is initiated with ubiquitination activation catalysed by an E1 enzyme, followed by the transfer of ubiquitin to the ubiquitin‐conjugating enzyme E2, and finally recognition of the targeted protein by an E3 ubiquitin ligase.^3^

Deubiquitinating enzymes (DUBs) reverse the action of ubiquitination by hydrolyzing ubiquitin molecules on ubiquitin‐linked proteins or precursor proteins, thereby inversely regulating protein degradation. The human genome encodes ~100 DUBs, making them the largest family of UPSs, and these DUBs can be classified into four categories according to their ubiquitin protease domains: ubiquitin‐specific peptidase (USP), ubiquitin C‐terminal hydrolase (UCH), Machado–Joseph disease protease (MJD) or otubain protease (OTU).

The role of the vast majority of DUBs have been identified, and they have been demonstrated to be related to diseases, including cancer,^4^ neurodegenerative diseases,^5^ brain diseases^6^ and infectious diseases.^7^ USPs, comprising the largest DUB superfamily with 58 members and accounting for about approximately of DUBs,^8^ have been found to play important roles in various cellular processes.^9^ In the past decade, USPs have attracted increasing attention as potential therapeutic targets for different diseases.^10^ In terms of bone metabolism, USPs have been found to be involved in the regulation of bone metabolism by promoting the osteogenesis of mesenchymal cells or by antagonizing the osteoclast differentiation of bone marrow monocytes.^11^, ^12^, ^13^, ^14^ Therefore, USPs are potential new targets in bone metabolism‐related disease mechanism research and drug development and show broad prospects for osteoporosis prevention and treatment.^13^ In this review, we provide the latest knowledge on the impact of these USPs on bone metabolism, including their activity in mesenchymal stem cells (MSCs), osteoblasts, osteoclasts, and osteocytes (Table 1).

**TABLE 1:** Effect of USPs in bone metabolism.

Species	USPs	Target protein	Signalling pathway	USPs‐affected Cell types	Function of USPs	References
Human	USP1	ID1 and ID2	BMP	Mesenchymal stem cell	Osteogenic differentiation (−)	[15]
Mouse	USP2	PTHR	PTH	Osteoblast	Proliferation (+)	[16]
Human	USP4	DVL	WNT/β‐catenin	Osteoblast	Differentiation (−)	[17]
Mouse	USP4	SIRT1	Sost	Osteocyte	Sclerostin transcription (−)	[18]
Mouse	USP6		BMP	Preosteoblast	Differentiation and maturation (−)	[19]
Human	USP7	SOX2 and NANOG		Mesenchymal stem cell	Self‐renewal capacity (+)	[20]
Mouse	USP7	KDM6B	BMP	Osteoblast	Differentiation and autophagy (+)	[21]
Human	USP7	RUNX2	BMP	Mesenchymal stem cell	Osteogenic differentiation (+)	[22]
Human	USP7			Adipose‐derived stem cells	Osteogenic differentiation (+)	[23]
Mouse	USP7	Axin	WNT/β‐catenin	Osteoblast	Differentiation (−)	[24]
Mouse	USP8	FZD5	WNT/β‐catenin	Osteoblast	Differentiation (+)	[25]
Mouse	USP10	p53	Oestrogen	Osteocytes and osteoblast	Senescence (+)	[26]
Human	USP11	MSX1	BMP	Mesenchymal stem cell	Osteogenic differentiation (+)	[27]
Mouse	USP13	PTEN	NF‐κB	Osteoclast	Differentiation (−)	[28]
Mouse	USP14	NLRC5	NF‐κB	Osteoclast	Differentiation (−)	[29]
Mouse	USP14	p53	p21	Osteoblast	Senescence (+)	[30]
Mouse	USP15	IκBα	NF‐κB	Osteoclast	Differentiation (−)	[31]
Mouse	USP15	ALK3	BMP	Myoblast progenitor	Osteogenic differentiation (+)	[32]
Mouse	USP15	β‐catenin	WNT/β‐catenin	Osteoblast	Differentiation (+)	[33]
Human	USP18	TAK1	NF‐κB	Monocytes and macrophages	Inflammatory response (−)	[34]
Mouse	USP18			Mesenchymal stem cell	Osteogenic differentiation (−)	[13]
Mouse	USP21			Mesenchymal stem cell	Osteogenic differentiation (+)	[13]
Human	USP25	TRAF6	NF‐κB	Osteoclast	Differentiation (+)	[14]
Mouse	USP26	β‐catenin	WNT/β‐catenin	Mesenchymal stem cell	Osteogenic differentiation (+)	[13]
Mouse	USP26	IκBα	NF‐κB	Bone myelomonocytes	Osteoclastogenesis (−)	[13]
Mouse	USP34	IκBα	NF‐κB	Osteoclast	Differentiation (−)	[12]
Human	USP34	Smad1 and RUNX2	BMP	Mesenchymal stem cell	Osteogenic differentiation (+)	[11, 35]
Human	USP53	β‐catenin	WNT/β‐catenin	Mesenchymal stem cell	Osteogenic differentiation (+)	[36]
Mouse	USP53		PTH	Mesenchymal stem cell	Osteogenic differentiation (−)	[37]
Mouse	USP53	VDR and Smad3	RANKL	Osteoblast and marrow adipocytes	Osteoclastogenesis (−)	[38]
Mouse	CYLD	SQSTM1/p62	NF‐κB	Bone marrow‐derived macrophages	Osteoclastogenesis (−)	[39]
					Differentiation (−)	
Mouse	CYLD	BCL3	NF‐κB	Bone marrow‐derived macrophages	Osteoclastogenesis (−)	[40]

*Note*: (+), Potentiation; (−), attenuation.

## 2 USPs: STRUCTURAL AND FUNCTIONAL DOMAINS

USPs constitute a cysteine protease family and they range from 50 to 300 kDa, with enzymatic activity realized through the thiol group of the central cysteine.^41^ The catalytic domain of a USP adopts a papain‐like fold, and this conserved catalytic domain enables most USPs to hydrolyze isopeptide bonds via a similar mechanism. Different from other DUBs, USPs carry specific cysteine protease domains that exhibit functional catalytic mechanisms; these domains comprise three conserved subdomains that resemble the relationship between the palm, thumb and fingers of a right hand.^42^ The catalytic site is located at the interface between the palm and thumb subdomains, while the finger subdomain is crucial for the interaction with a distal ubiquitin.^42^ Most members of the USP family include a core catalytic domain and other N‐ and C‐terminal extensions, and they may be composed of different domains or sequences with specific functions.^43^

Ubiquitin is a small conserved regulatory protein comprising 76 amino acids and can be linked to methionine (M1) residues and lysine (K) residues, such as K6, K11, K27, K29, K33, K48 and K63.^44^ Notably, K48 and K63 chains have been the most extensively studied ubiquitin chains, with the K48 linked ubiquitin chain found to be associated with ATP‐dependent proteasome degradation,^45^ while the K63 ubiquitin chain plays a role in signal transduction and DNA repair.^10^, ^46^ Most USPs show hydrolytic activity on K48‐, K63‐ and Met1‐linked ubiquitin chains and participate in the regulation of various biological processes, such as cell migration, tumorigenesis, immune responses, and inflammation.^44^, ^47^ The abnormal expression or mutation of USPs usually leads to a series of diseases^48^ (Table 2), including bone metabolism disorders, such as osteoporosis.^3^

**TABLE 2:** Diseases associated with USPs mutation.

USPs mutation	Diseases	References
USP1	Fanconi anaemia	[49]
USP3	Crohn disease	[50]
USP6	Nodular fasciitis	[51]
	Aneurysmal bone cyst	[52]
USP7	UV‐sensitive syndrome	[53]
	Hao–Fountain syndrome	[54
USP8	Cushing disease	[55]
USP9x	Multiple epiphyseal dysplasia	[56]
	USP9X‐female syndrome	[57]
USP16	Down syndrome	[58]
USP18	Multiple sclerosis	[59]
	Pseudo‐TORCH syndrome	[60]
USP24	Parkinson disease, late‐onset	[61, 62]
USP26	Male Infertility	[63]
USP36	Primary ovarian insufficiency	[64]
	Type 2 diabetes	[65]
USP38	Bronchial asthma	[66]
USP40	Crohn disease	[50]
	Parkinson disease, late‐onset	[62]
USP42	Acute myeloid leukaemia	[67]
USP44	Congenital heart disease	[68]
	Intellectual disability	[69, 70]
USP45	Leber congenital amaurosis	[71]
USP47	Amyotrophic lateral sclerosis	[72]
USP48	Crohn disease	[73]
USP53	Hereditary cholestasis	[74, 75]
	Progressive hearing loss	[76]
CYLD	Crohn disease	[50]

## 3 EFFECTS OF USPs ON THE SELF‐RENEWAL AND OSTEOGENIC DIFFERENTIATION OF MSCs

**FIGURE 1:**
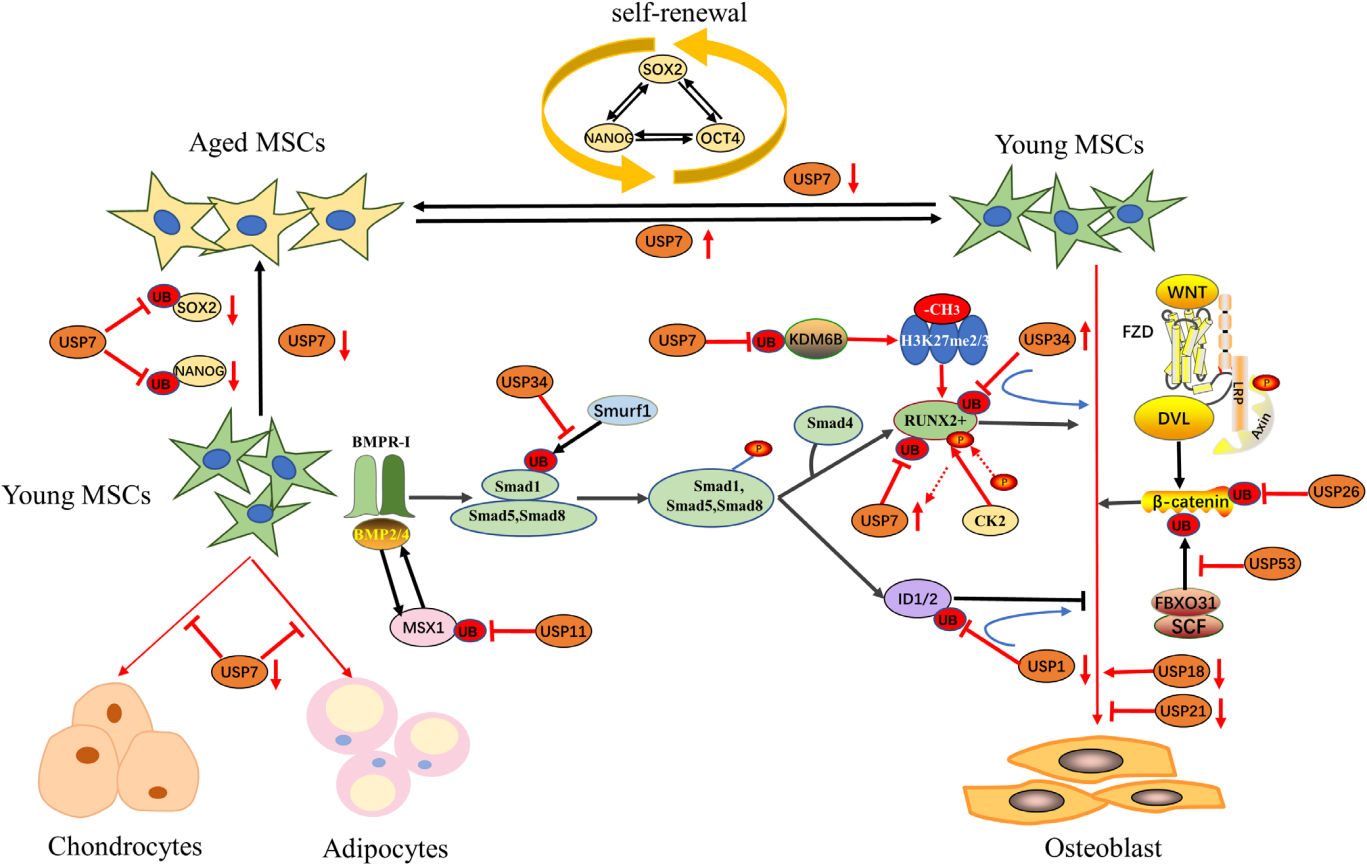
Effect of USPs on the self‐renewal and osteogenic differentiation of MSCs. USP7 enhances the self‐renewal capacity of BMSCs via the deubiquitination and stabilization of SOX2 and NANOG and is necessary in the early stages of the osteogenic, adipogenic and chondrogenic differentiation of BMSCs. USP7 also regulates BMP signalling pathway through deubiquitination and stabilization of KDM6B and RUNX2+, thus participating in osteogenic differentiation of BMSCs. In addition, USP34 interacts with Smad1 and RUNX2+, USP1 interacts with ID1/2, and USP11 interacts with MSX1 to inhibit their ubiquitination degradation and then regulate BMP signalling pathways. USP26 and USP53 regulate WNT/β‐catenin signalling by inhibiting the ubiquitination degradation of β‐catenin. USP18 and USP21 are also involved in osteogenic differentiation of MSCs. However, the specific mechanism remains unknown.

Mesenchymal stem cells (MSCs) are multipotent progenitors with self‐renewal capability and multidirectional differentiation potential; that is, they can undergo osteogenesis, adipogenesis and chondrogenesis.^77^ Osteoblasts differentiated from MSCs are the main sources of bone formation, and disruption to the MSC‐osteoblast differentiation process leads to improper bone formation and imbalanced bone homeostasis.^78^ Increasing evidence had indicated that UPS is essential for fine‐tuned regulating of MSC differentiation,^79^ with E3 ligases ubiquitinating self‐renewal‐associated regulatory proteins to promote stem cell differentiation, while DUBs stabilize proteins to promote cell self‐renewal and stemness.^80^ As the most extensively studied DUBs, several USPs, such as USP1,^15^ USP7,^20^ USP11,^27^ USP15,^32^ USP26,^13^ USP 34^81^ and USP53,^36^ have been found to regulate the self‐renewal and osteogenic differentiation of MSCs (Figure 1).

SOX2 and NANOG are two proteins necessary for the self‐renewal and pluripotency of stem cells.^82^ USP7, also called herpes virus‐associated ubiquitin‐specific protease (HAUSP), modulates the self‐renewal capacity of bone marrow MSCs (BMSCs) via the deubiquitination and stabilization of SOX2 and NANOG and is necessary in the early stages of the osteogenic, adipogenic and chondrogenic differentiation of hBMSCs.^20^ Interestingly, the downregulation of USP7 significantly inhibited the osteogenic, adipogenic and chondrogenic differentiation potential of BMSCs, while USP7 overexpression exerted no significant impact on the differentiation of BMSCs. Growth arrest is required for cells to undergo differentiation.^83^ However, USP7 overexpression increased cell proliferation, which may be the reason that overexpressed USP7 did not enhance the differentiation of the BMSCs.^20^ Another study, from Tang et al, showed that USP7 regulated not only the osteogenic differentiation of human BMSCs but also the osteogenic and adipogenic differentiation of adipose‐derived stem cells.^23^ However, the specific mechanism by which USP7 regulates the osteogenic differentiation of BMSCs is still unknown. Casein kinase 2 (CK2), a highly conserved serine/threonine kinase, is a negative regulator of BMP signalling and osteoblast differentiation.^84^, ^85^ CK2 phosphorylates RUNX2 to recruit the deubiquitinase USP7, which stabilizes the RUNX2 level by protecting it from ubiquitin‐dependent proteasomal degradation.^22^ Therefore, USP7 is likely involved in the osteogenic differentiation of MSCs via the deubiquitination and stabilization of RUNX2.^22^ In addition, USP7 deubiquitinated and stabilized the expression of K‐specific demethylase 6B (KDM6B) to promote the proliferation, differentiation and autophagy of osteoblasts.^21^ KDM6B, a histone 3 lysine 27 (H3K27) demethylase, plays critical roles in the osteogenic commitment of MSCs by catalysing the demethylation of H3K27me2/3 and indirectly controlling RUNX2 expression by regulating BMP and HOX.^86^ Similar to USP7, USP34 positively regulate the osteogenic differentiation of MSCs. In addition, USP34 simultaneously stabilizes the expression of Smad1 and RUNX2.^81^ Specific deletion of USP34 in MSCs inhibits its osteogenic differentiation and thus impairs fixation of titanium implants in mice.^35^

Bone morphogenetic protein (BMP) is an important signalling factor in osteogenesis. Inhibitors of DNA binding proteins (IDs) are prominent targets of BMP signalling,^87^ and they can inhibit differentiation and maintain stem cell fate.^15^ During the osteogenic differentiation of MSCs, ID expression is tightly regulated, with rapid upregulation that promotes cell proliferation and inhibits the differentiation of early progenitor cells, thereby maintaining stem cell fate. Later, the ID expression level returns to the basal level, enabling the terminal differentiation of osteoblasts.^88^ ID1, ID2 and ID3 are ubiquitinated via k48 linkages and subsequently degraded by the 26S proteasome. The DUB USP1 has been shown to be expressed in primary BMSCs and to impair osteogenic differentiation of MSCs via the deubiquitination and stabilization of the ID1 and ID2 proteins.^15^ However, USP1 levels have been shown to decline steadily during differentiation into osteoblasts, which explains the reason that ID protein expression returns to basal level in the later differentiation stage.^15^ Furthermore, the USP1 inhibitor ML323 enhances the osteogenic potential of human dental pulp stem cells.^89^ Msh isoform 1 (MSX1) is also an upstream and downstream regulator of the BMP signalling pathway, and a balanced MSX1 protein level is crucial to normal osteogenesis.^90^ USP11 prevents MSX1 protein degradation via its DUB activity, making it a positive regulator of BMSC osteogenic differentiation.^27^

Wnt/β‐catenin is a crucial regulatory signalling pathway involved in the osteogenic differentiation of BMSCs.^91^ As a member of the FBP family, FBXO31 interacts with the SCF complex to form the functional E3 ubiquitin ligase SCF‐FBXO31, which regulates the ubiquitination of target proteins. SCF‐FBXO31 negatively regulates the osteogenic differentiation of BMSCs via its E3 ubiquitin ligase activity, leading to the degradation of β‐linked proteins.^36^ USP53 interacts with FBXO31 to inhibit the ubiquitination and degradation of β‐catenin, thereby activating Wnt/β‐catenin signalling to regulate the osteogenic differentiation of BMSCs.^36^ However, another study, from Hariri et al.,^37^ showed the opposite result, in which Usp53 knockdown in murine BMSCs enhanced osteogenesis and inhibited adipogenesis. The main differences between the two studies were the sources of the BMSCs, indicating that USP53 may have played opposite roles in the osteogenic differentiation of human and mouse BMSCs. The possible explanation for this discrepancy may be the differences protein sequences between human and mouse that may result in different protein interactions and functions in each species.^3^

USP26 is a recently identified modulator of bone homeostasis that coordinates both bone formation and resorption.^13^ The expression of USP26 was downregulated in MSCs from aged mice, bone myelomonocytes (BMMs) and MSCs from ovariectomized mice, indicating that ageing and oestrogen deficiency may be involved in regulating USP26 expression. In contrast, USP26 supplementation attenuated bone defects in the aged mice and restored bone tissue after ovariectomy‐induced bone loss. Mechanistically, USP26 not only enhanced the osteogenic differentiation of MSCs via the deubiquitination and stabilization of β‐catenin but also inhibited the osteoclastic differentiation of BMMs by stabilizing the inhibitor of NF‐κBα (IκBα).^13^ In addition, this study showed that depletion of USP26 impaired MSC chondrogenesis, suggesting that USP26 may be involved in osteoarthritis (OA) development. However, the mechanism of USP26 action still needs to be explored. The study also reported that downregulation of USP18 significantly enhanced the osteogenic potential of MSCs, while USP21 depletion inhibited both the osteoblast differentiation of MSCs and the formation of multinucleated osteoclasts derived from BMMs.^13^

## 4 EFFECTS OF USPs ON OSTEOBLASTOGENESIS AND BONE FORMATION

**FIGURE 2:**
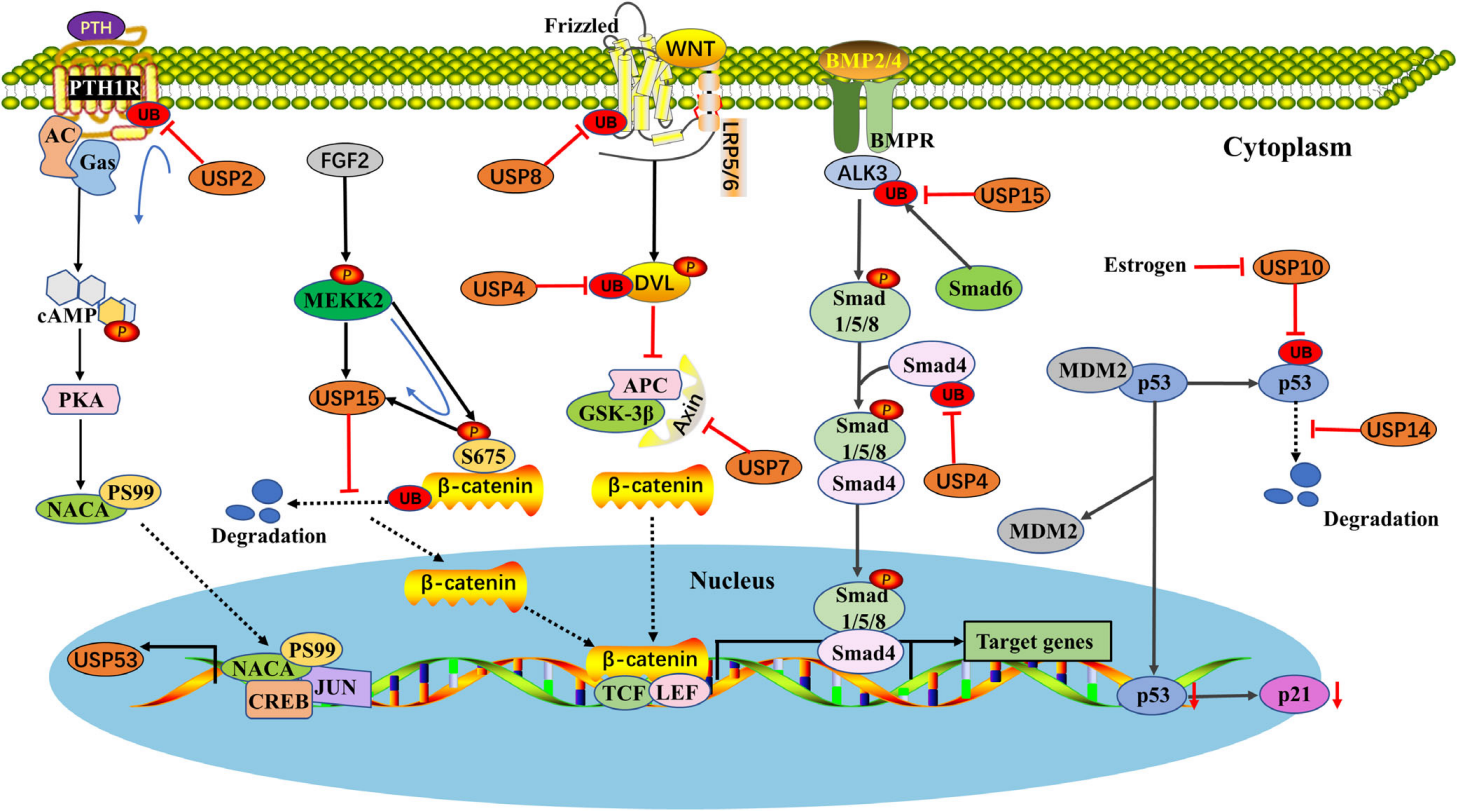
Effect of USPs on osteoblastogenesis and bone formation. USPs regulates the proliferation, differentiation and maturation of osteoblasts via a variety of signalling pathways. USP2 protected PTHR from PTH‐induced downregulation via direct deubiquitination of the receptor. USP4, USP7, USP8 and USP15 participate in the WNT/β‐catenin by interacting with DVL, Axin, WNT and β‐catenin and inhibiting their ubiquitination degradation, respectively. In addition, USP4 and USP15 can also participate in BMP signalling by interacting with Smad4 and ALK3 and inhibiting their ubiquitination degradation, respectively. USP10 and USP14 act as senescence inducers by stabilizing p53 expression and then promote the transcription of p21.

In the skeletal system, osteoblasts are the main functional cells of bone formation, responsible for the synthesis, secretion and mineralization of bone matrix.^92^ The proliferation, differentiation and maturation of osteoblasts are regulated by a variety of signalling pathways, such as PTH, WNT/β‐catenin, BMP and Oestrogen, which are also regulated by USPs (Figure 2).

Parathyroid hormone (PTH) is an effective regulator of bone metabolism that effects osteoblast proliferation through the G protein‐coupled transmembrane receptor PTH1R.^93^ Adenylate cyclase (AC) is activated to produce cAMP, and then, protein kinase A (PKA) is activated. PKA phosphorylates the alpha chain of the nascent polypeptide‐associated complex αNAC (NACA) at serine residue 99 (S99) and induces its nuclear translocation, subsequently increasing the transcription of osteogenic target genes.^37^ On the cell membrane of osteoblasts, the PTH (amino acid [aa] 1–84) and PTH (aa 1–34) peptides bind and activate PTHR. In addition, PTH (aa 1–34) promoted PTHR ubiquitination and deubiquitination coupling.^94^ USP2, also known as UBP41, has been found to be essential for PTH (aa 1–34)‐induced osteoblast proliferation.^16^, ^95^ Studies have shown that PTH (aa 1–34) induced USP2 expression through its direct effect on the cAMP pathway,^16^ and USP2 protected PTHR from PTH‐induced downregulation via direct deubiquitination of the receptor, thereby promoting receptor recycling.^94^ Moreover, the main role played by USP2 in osteoblast proliferation is to promote a balance between the rapid deubiquitination and recycling of the PTH receptor.^16^, ^94^ USP53 is also a PTH target in osteoblasts. However, the effect of USP53 expression levels on PTH responses varies depending on the cell maturation stage during osteoblastogenesis.^37^ In the nucleus, NACA binds to the USP53 promoter and functions with JUN/CREB to enhance USP53 transcription in osteoblasts^3^.

The canonical WNT/β‐catenin signalling pathway plays indispensable roles in osteoblast differentiation and bone formation.^96^ WNT ligands first bind to their cell surface receptors low‐density lipoprotein receptor‐related protein (LRP5/6) and Frizzled (FZD) to recruit, phosphorylate and activate the cytoplasmic protein dishevelled (Dvl). The phosphorylation and degradation of β‐catenin is inhibited by the dissociation of GSK‐3b from Axin, which ultimately stabilizes β‐catenin, promoting its translocation into the nucleus where it induces the transcription of osteogenesis‐related genes.^2^, ^97^ USP8 stabilizes FZD expression through deubiquitination and ensures WNT‐induced osteogenesis.^98^ USP4 inhibits osteoblast differentiation and bone formation by inhibiting Wnt/β‐catenin signalling via the removal of K63‐linked polyubiquitin chains from Dvl.^99^ USP7 directly interacts with the Axin through its TRAF domain and promotes Axin deubiquitination and stabilization, thereby inhibiting Wnt/β‐catenin signalling to regulate osteoblast differentiation.^24^ Kcnma1, the gene encoding the BK channel, can upregulate the protein level of USP7 and then promote the degradation of β‐catenin in osteoblasts by stabilizing Axin, thereby inhibiting osteoblast differentiation and proliferation.^98^ Mitogen‐activated protein kinase kinase 2 (MEKK2) has been found to mediate β‐catenin activation in osteoblasts via a mechanism that differs from that of the canonical WNT pathway.^33^ FGF2 activates MEKK2 to phosphorylate β‐catenin at serine 675 and promotes the recruitment of the DUB enzyme USP15. In turn, USP15 enhances WNT signalling by preventing basal turnover of β‐catenin via the inhibition of its ubiquitin‐dependent proteasomal degradation.^33^

BMP2 is a major extracellular signalling molecule that induces osteoblast differentiation and promotes bone formation. BMP‐2 binds to cell surface receptors leading to the phosphorylation of intracellular transduction factors Smad1/5/8, which in turn interact with Smad4, and Smad4 is then translocated into the nucleus, where is interact with Runx2 to induce osteogenic gene expression.^100^ In mouse embryonic stem cells, USP4 is a potent activator of BMP signalling that acts via the direct deubiquitination of R‐Smad‐Smurf2‐induced monoubiquitination of Smad4.^99^ BMP2 treatment increased the expression of the early osteoblast marker alkaline phosphatase, but this induction was prevented by the depletion of USP15, which is also a DUB of receptor‐activated Smad.^101^ Smad6, a transcriptional target of BMP, negatively regulates the BMP pathway by recruiting the E3 ubiquitin ligase Smurf1/2 to target and degrade the type I BMP receptor ALK3, thereby inhibiting the phosphorylation and activation of Smad1/5/8.^102^, ^103^ As an interactor of Smad6 and ALK3, USP15 can interact with ALK3 and stabilize ALK3 expression through its DUB activity, which then enhances BMP‐induced Smad1 phosphorylation.^32^ USP6, also known as TRE17, has been studied primarily in aneurysmal bone cysts.^104^ USP6 potently inhibits the maturation of preosteoblasts via an autocrine mechanism involving BMP‐4 dysregulation and the upregulation of the BMP antagonist Gremlin‐1.^19^ However, the mechanism by which USP6 regulated BMP action remains unclear.

Oestrogen also plays an important role in maintaining bone homeostasis. Recently, many investigators have shown an interest in the potential role of oestrogen in mediating cellular senescence, a key process in age‐related diseases and bone loss.^26^, ^105^, ^106^ The transcription factor p53 has been associated with cellular senescence and apoptosis and is a negative regulator of bone regeneration.^107^ USP10, which can act as a senescence inducer,^108^ promotes p21 transcription by directly removing ubiquitin molecules from p53 to maintain p53 stability and function.^109^, ^110^ Oestrogen increased the protein levels of p53 and p21 in MC3T3‐E1 cells, mainly through a USP10‐dependent deubiquitination pathway, to mediate p53 stability.^26^ USP14 is also a potential DUB enzyme that acts on p53, and USP14 may contribute to dexamethasone‐induced apoptosis in MC3T3‐E1 cells by stabilizing p53 expression.^30^

## 5 EFFECTS OF USPs ON OSTEOCLASTOGENESIS AND BONE RESORPTION

**FIGURE 3:**
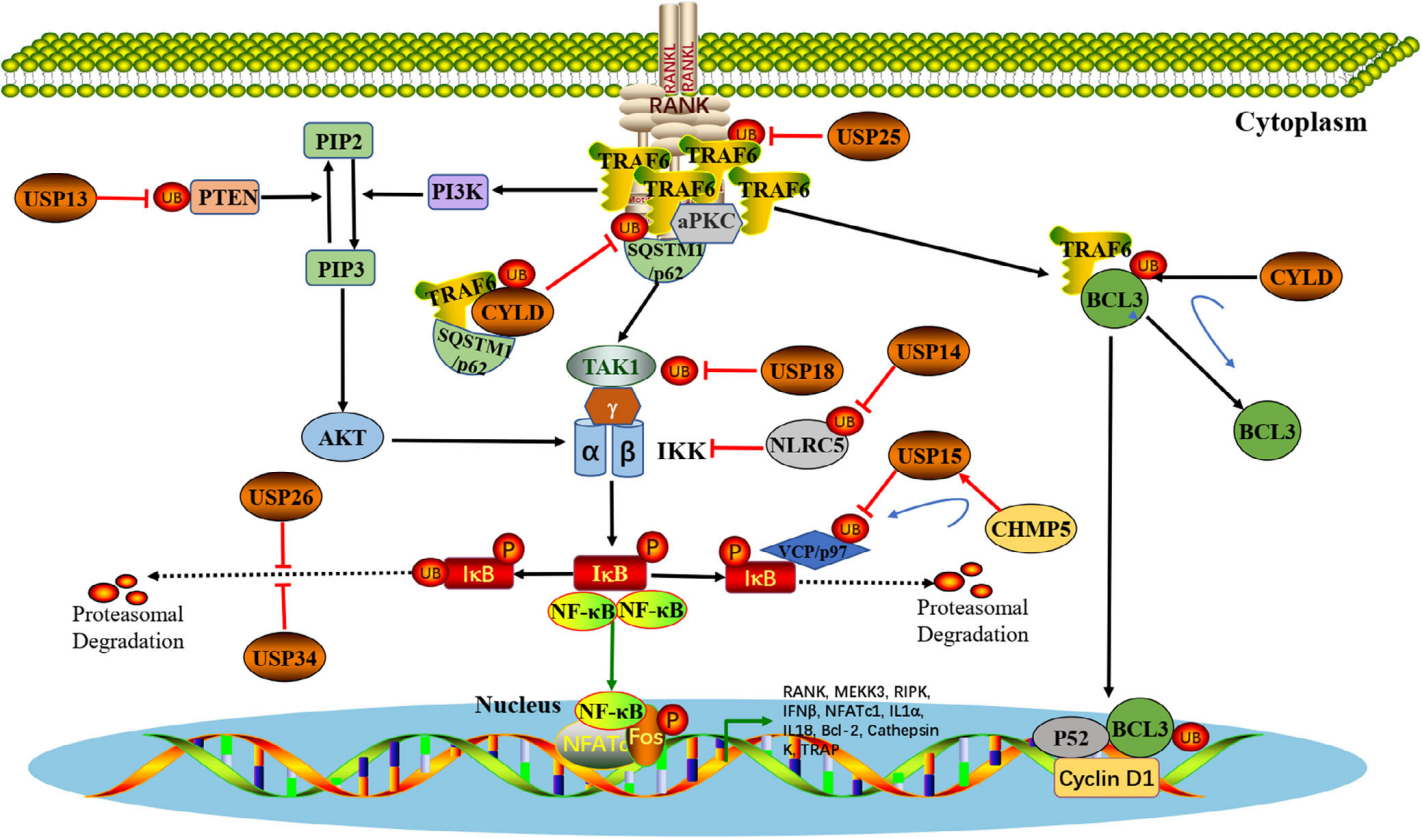
Effect of USPs on osteoclastogenesis and bone resorption. USPs are involved in the regulation of osteoclastogenesis and bone resorption mainly through NF‐κB signalling pathway. TRAF6 recruits SQSTM1/p62 to form a ternary complex with aPKC and then activates NF‐κB signalling. CYLD physically interacts with SQSTM1/p62 and is therefore recruited into TRAF6 to negatively regulate RANK signalling by antagonizing TRAF6‐mediated polyubiquitination. CYLD can also remove the polyubiquitin chain of BCL3 by interacting with TRAF6, leading to downregulation of the transcription of the BCl3‐dependent cyclin D1 gene. In addition, USP25 and USP18 may activate the NF‐κB signalling pathway via deubiquitination and stabilization of TRAF6 and TAK1 expression, respectively. USP14 inhibits IKK by stabilizing NLRC5, while USP13 reduces AKT and IKK levels by rescuing and stabilizing PTEN, both of which inhibit NF‐κB signalling. Finally, USP15, USP26 and USP34 interact with IκBα to inhibit NF‐κB signalling via their deubiquitination and stabilization of IκBα, thereby inhibiting osteoclastogenesis.

Osteoclasts are multinucleated cells generated from monocyte/macrophage precursor cells through a progressive differentiation process involving regulation by cytokines and transcription factors related to bone resorption and remodelling.^111^, ^112^ TRAF6 is a major adapter protein that mediates the signalling cascade after RANKL–RANK activation in osteoclasts.^113^ TRAF6 binds to RANK to recruit and activate TGF‐beta‐activated kinase 1 (TAK1) and then activates inhibitor of nuclear factor kappa‐B (IκB) kinase (IKK).^112^ The IκB protein is subsequently phosphorylated, ubiquitinated and degraded to activate NF‐κB. The active NF‐κB protein is then transferred to the nucleus where it initiates the transcription of osteoclast‐specific genes and ultimately induces the differentiation of osteoclast precursors into mature osteoclasts.^114^ USPs also plays an important role in the proliferation, differentiation and maturation of osteoclasts (Figure 3).

Our previous study showed that the expression of USP25 in peripheral blood monocytes is negatively correlated with bone mineral density (BMD) and positively correlated with TRAF6 expression in females, suggesting that USP25 may participate in the differentiation of monocytes into osteoclasts by stabilizing the expression of TRAF6.^14^ SQSTM1/p62 (sequestosome 1) is a multifunctional ubiquitination‐bound adaptor protein involved in the regulation of autophagy.^115^ TRAF6 recruits SQSTM1/p62 to form a ternary complex with atypical protein kinase C (aPKC) and then activates NF‐κB signalling.^116^ The deubiquitination enzyme Cylindromatosis (CYLD) physically interacts with SQSTM1/p62 and is therefore recruited into TRAF6 to negatively regulate RANK signalling by antagonizing TRAF6‐mediated polyubiquitination.^39^ The TRAF6‐p62‐CYLD signalling complex is an important link in the NF‐κB pathway of osteoclast differentiation and is currently an important target for developing new strategies for treatments of bone diseases.^117^, ^118^, ^119^, ^120^, ^121^, ^122^ B‐cell Chronic lymphocytic leukaemia protein 3 (BCL3) is another protein that physically interacts with TRAF6 and is involved in non‐canonical NF‐κB signalling pathway. CYLD is assembled into TRAF6, which then removes the K63‐linked polyubiquitin chain of BCL3, which is then transported out of the nucleus, resulting in downregulation of transcription of the BCl3‐dependent cyclin D1 gene.^40^ Other USPs, such as USP8^115^ and USP36^123^ were also found to deubiquitinate SQSTM1/p62 and suppress its autophagy activity, and USP20 was found to regulate the TNFα‐Induced NF‐κB Signalling Pathway by stabilizing the SQSTM1/p62 protein.^124^ However, their role in bone metabolism have not yet been extensively studied, and further study will deepen the understanding of the relationship between autophagy and osteoclasts.

USP18, also known as UBP43, was originally identified as a type I interferon‐responsive gene.^125^ Yim et al. found that RANKL treatment induced the expression of USP18 in preosteoclast cells, and depletion of USP18 increased RANKL‐mediated osteoclastogenesis, although the mechanism remains unclear.^126^ TAK1‐mediated activation of NF‐κB signalling has been shown to require K63‐linked ubiquitination of TAK1.^127^, ^128^ USP18 can cleave the TAK1 K63‐linked polyubiquitin chain^34^, ^129^ and downregulate TAK1‐regulated NF‐κB signalling in BMMs,^129^ which may explain the reason that USP18 deficiency increases RANKL‐mediated osteoclastogenesis. Charged multivesicular body protein 5 (CHMP5) is a novel modulator of the NF‐κB pathway.^130^ CHMP5 is an adapter that recruits USP15 to the VCP/p97 complex and reverses the ubiquitination of the VCP/p97 client protein IκBα through the DUB activity of USP15, resulting in the retention of NF‐κB in inactive cytoplasmic complexes and inhibition of RANK‐mediated NF‐κB activation.^31^ In addition, USP26^13^ and USP34^12^ can also interact with IκBα to inhibit NF‐κB signalling via their deubiquitination and stabilization of IκBα, thereby inhibiting osteoclastogenesis.

PTEN has been identified as an important regulator in RANKL‐induced osteoclastogenesis and participates in the regulation of the phosphorylated inositol 3‐kinase (PI3K)/AKT pathway through the dephosphorylation of phosphatidylinositol 3,4,5‐triphosphate (PIP3).^131^, ^132^ AKT directly phosphorylates and activates IKKs to promote IκB degradation, thereby activating the NF‐κB pathway.^133^ Huang et al.^28^ found that USP13 directly interacted with PTEN and inhibited its ubiquitination‐related degradation. Overexpression of USP13 rescued and stabilized PTEN protein level, which helped to reduce AKT and NF‐κB signalling activation, thereby inhibiting the osteoclastogenesis induced by RANKL. In addition, overexpression of USP13 also increased serum OPG levels, decreased RANKL levels in synovial tissues, and attenuated bone destruction in mice with collagen‐induced arthritis (CIA).^28^ Proinflammatory NF‐κB and PI3K/AKT signalling has also been shown to be among the most important contributors to induce particle‐induced inflammatory reactions in macrophages and bone cell lysis.^133^, ^134^, ^135^ NOD‐like receptor family CARD domain‐containing 5 (NLRC5), a negative regulator that blocks IKKα and IKKβ activity, inhibits the activation of the PI3K/AKT and NF‐κB signalling pathways.^136^ USP14, a major proteasome regulator,^137^ can enhance the NLRC5‐mediated inhibition of NF‐κB signalling by reversing TRAF2/6‐mediated NLRC5 ubiquitination.^138^ Activation of the USP14‐NLRC5 pathway inhibited proinflammatory cytokine expression, PI3K/AKT and NF‐κB signalling activities, M1 polarization of macrophages and osteoclast formation, thereby inhibiting titanium particle‐induced osteolysis.^29^

## 6 EFFECT OF USPs ON THE REGULATION OF OSTEOBLAST–OSTEOCLAST COUPLING

The mutual regulation between osteoblasts and osteoclasts is the basis of bone formation and bone resorption balance during bone remodelling.^139^ Paracrine action of cytokines and secreted molecules is one of the main ways of osteoblast–osteoclast coupling.^140^ RANKL, which is secreted mainly by osteoblasts and osteocytes in bone, is a major regulator of osteoclastogenesis. A recent study by Hariri et al.^38^ found that USP53 is involved in regulating osteoblast‐dependent osteoclastogenesis by controlling the expression of RANKL. Mechanistically, USP53 inhibits the interaction between Smad3 and vitamin D receptors (VDR), resulting in reduced binding of VDR to its cognate elements, thereby inhibiting the activation of RANKL.^38^ In addition, it also found that Smurf2 can regulate the ubiquitination of Smad3 and affect the expression of RANKL in osteoblasts by inhibiting the interaction between Smasd3 and VDR.^141^ Therefore, USP53 may promote the monoubiquitination of Smad3 by binding and stabilizing Smurf2 or another relevant E3 ligase, thereby inhibiting its interaction with VDR and subsequently inhibiting the activation of RANKL. Currently, the role of USPs in the regulation of osteoblast–osteoclast coupling is poorly studied. Future studies should find more USPs that are involved in regulation of osteoblast–osteoclast coupling.

## 7 EFFECTS OF USPs ON OSTEOCYTES AND BONE REMODELLING

Osteocytes are the main cells in mature bone tissue, accounting for about 95% of the total bone cells.^142^ Osteocytes can secrete paracrine factors, such as OPG, RANKL and sclerostin (Sost), which can participate in the regulation of osteoblast and osteoclast differentiation and activity and play a key role in bone remodelling.^143^, ^144^ Sost binds to LPR5/6 on osteoblast cell membranes to interfere with WNT binding to WNT receptor Frizzled, which acts as an antagonist of WNT/β‐catenin signalling to inhibit osteogenesis and bone formation.^145^ Sirtuin 1 (SIRT1) inhibits the expression of Sost by modifying the acetylation of H3K9 at the Sost promoter.^146^, ^147^ The phosphorylation of USP4 by casein kinase 2 (Ck2) inhibits the ubiquitination degradation of SIRT1, thus inhibiting the transcription of Sost and reducing the RANKL/OPG ratio in osteocytes, thereby reducing osteoclastogenesis and increasing osteoblastogenesis.^18^ Overall, Sost expression regulated by CK2–USP4–SIRT1 pathway is essential for osteocytes to maintain bone homeostasis.

**FIGURE 4:**
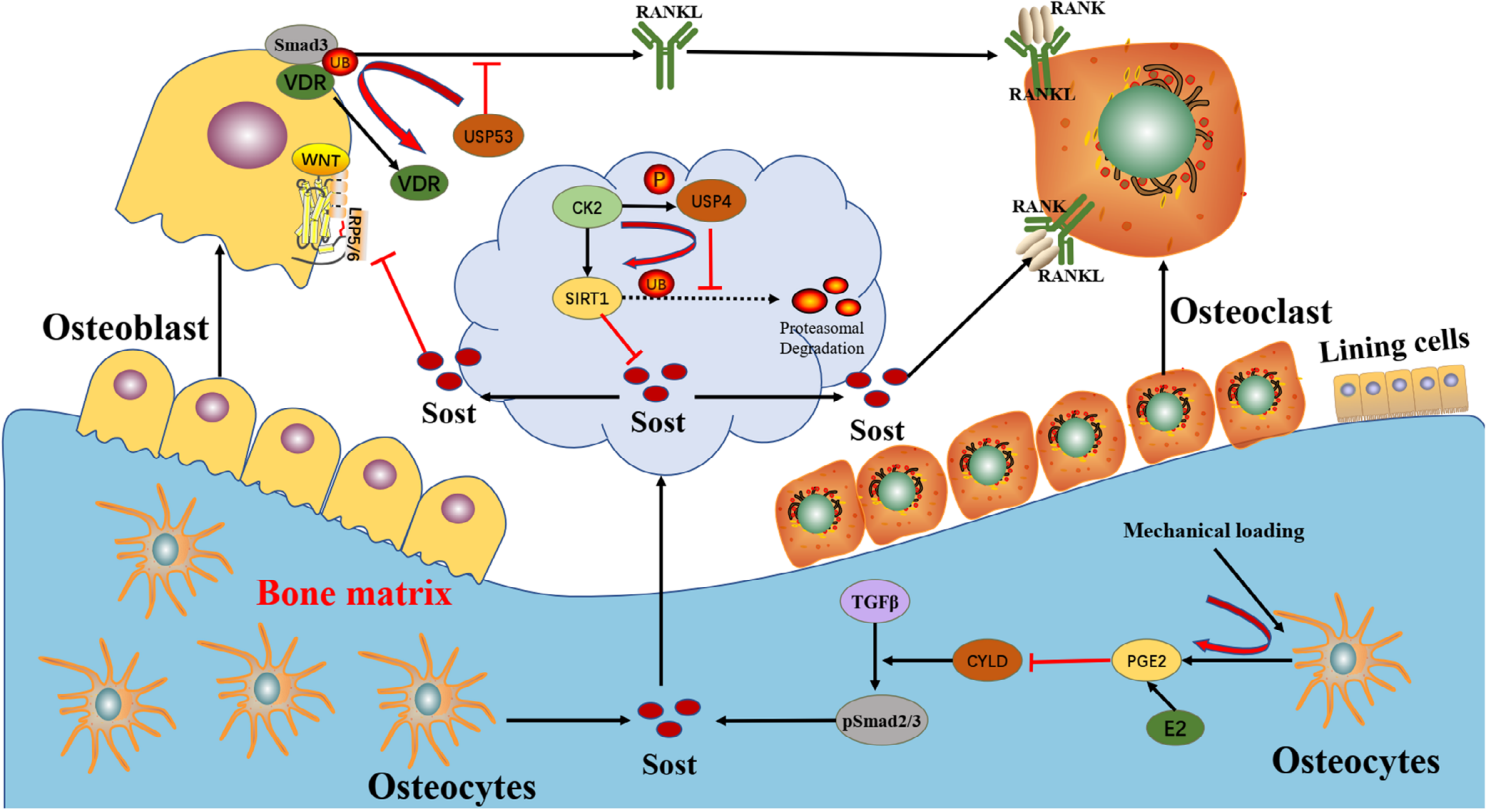
Effect of USP4 secretion on sclerostin in osteocyte. Mechanical loading up‐regulates the expression of PGE2, which binds to EP2 to inhibit TGFβ signalling through a CYLD‐dependent proteasome mechanism, thereby inhibiting the transactivation of pSmad2/3 and subsequently inhibiting the expression of Sost. The phosphorylation of USP4 by Ck2 can also inhibit the transcription of Sost by inhibiting ubiquitination degradation of SIRT1. Sost then acts as an antagonist of WNT/β‐catenin signalling and a promoter of RANK/RANKL signalling to modulate bone remodelling. In addition, USP53 is involved in the regulation of osteoblast–osteoclast coupling. Mechanistically, USP53 inhibits the interaction between Smad3 and VDR in osteoblasts, resulting in reduced binding of VDR to its cognate elements, thereby inhibiting the activation of RANKL.

Mechanical loading is also an important factor for the maintenance of bone mass. Osteocytes are bone‐embedded cells that sense mechanical stimulation and respond by regulating the activity of osteoblasts and osteoclasts.^148^ Prostaglandin E2 (PGE2) is rapidly upregulated in osteocytes upon mechanical stimulation. PGE2 acts through its receptor (EP2) and inhibits TGFβ signalling through a proteasome mechanism dependent on deubiquitinase CYLD, thereby inhibiting pSmad2/3 and its transactivation of Serpine1 in osteocytes.^149^ The downregulation of Smad3 phosphorylation and activity thereby inhibited the expression of Sost in osteocytes.^150^ Thus, mechanical loading maintains bone homeostasis through the TGFβ pathway to regulate Sost expression (Figure 4).

## 8 CONCLUSION

Recent studies and evidence continue to reveal the involvement of USPs as DUBs in bone metabolic processes (Table 1). In this review, we highlight the role of USPs as regulators of signalling outcomes and the differentiation fates of MSCs, osteoblasts, osteoclasts and osteocytes. Abnormal expression of USPs is an important factor leading to an imbalance between bone formation and bone resorption. Further mechanistic studies into USP‐mediated regulation of osteoblastogenesis, osteoclastogenesis and osteoblast–osteoclast coupling will be pivotal for the discovery and development of novel USP‐targeting therapeutic strategies for osteoporosis.

## AUTHOR CONTRIBUTIONS

**Jianlin Shen:** Conceptualization, Writing ‐ original draft. **Xiaoning Lin:** Conceptualization, Writing ‐ original draft. **Feifei Dai:** Conceptualization, Writing ‐ original draft. **Guoli Chen:** Review & editing. **Haibin Lin:** Review & editing. **Bangjiang Fang:** Supervision, Writing ‐ review & editing. **Huan Lin:** Supervision, Writing ‐ review & editing.

## FUNDING INFORMATION

This study was supported by the Natural Science Foundation of Fujian Province (2020J011260 to Jianlin Shen), Research Foundation Project of Putian University (2019070 to Jianlin Shen), and Science and Technology Project of Putian City (2060499 to Jianlin Shen).

## CONFLICT OF INTEREST STATEMENT

The authors declare no conflicts of interest.

## Data Availability

Data sharing is not applicable to this article as no new data were created or analyzed in this study.
